# The Annual Address Delivered before the Niagara County Medical Society, June 7th, 1870

**Published:** 1870-09

**Authors:** A. W. Tryon

**Affiliations:** President


BUFFALO

### Medical and Surgical Journal.

VOL. X. SEPTEMBER, 1870. No. 2.

### Original Communications.

ART. I.—*The Annual Address delivered before the Niagara County Medical Society, June 7th,* 1870, *by* A. W. TRYON, M. D., *President.*

(PUBLISHED BY REQUEST OF THE SOCIETY.)

MR. VICE-PRESIDENT AND MEMBERS OF THE SOCIETY:

Time, in his onward flight, has again brought the day of our annual meeting; and, according to the requirements of the by-laws of this organization, the duty devolves upon the retiring President to address the society before vacating his chair for his successor.

The cyclical year of our association, that is brought to a close by our meeting of to-day, may be called a very good one. Certainly the society has cause for thankfulness in not being called to mourn the absence of any of its members by death. The "grim monster" has not ventured to enter our circle. Though time is flinging out the white flag of truce from full many a *pall*, yet, with feelings of gratitude, do we still observe the full vigor of action, and the firmness of footstep of all our members. May the coming year be as merciful to us, and when the next annual meeting shall take place, may the same feelings of joy over an unbroken circle prevail.

The past year has been one of general healthfulness. No devastating epidemic has been permitted to spread sorrow in our midst. Yet it has not been so pitifully healthy that the doctors have suffered materially.

There has been performed in this city, during the past year, several of the major operations of surgery, one of which, for its success, deserves at least a passing notice. It was the removal of a goitrous gland from the neck of Miss Merinda Davis. This tumor had been many years in growing, and had become of such monstrous size as to seriously threaten life. The removal of it, it seemed, would inevitably cause death from hemorrhage, either primary or secondary. In fact, all medical advisers discouraged the operation. But the heroic lady resolved upon having it done; and Professor WILLIAM WARREN GREEN, of Portland, Maine, was selected to perform the operation. Miss Davis, with unexampled fortitude, fully realizing all the peril of life in the case, after commending her soul to God, laid herself upon the operating table with a calm resignation to her fate, most beautiful to behold, because of this implicit trust in a higher Power, that "holdeth us, as it were, in the hollow of His hand," and "doeth all things well." The tumor was successfully removed, and by faithful and careful after attention, the lady is blessed with a full recovery. Thanks to the skill of the honored member of the profession, who so skillfully performed the operation. Thanks to the lady's noble fortitude and heroic courage; the highest thanks to Him who keeps his promise to hear, and answer the believing prayer, another laurel chaplet is laid upon the altar of our profession—another pure and holy life has been prolonged, and made of greater usefulness upon the earth.

There are other items of practice and experience that your speaker would like to recount, believing they would be of general interest but he must hasten to what he desires to be the subject of his address.

### THE STANDARD OF THE MEDICAL PROFESSION.

There has been enough written to convince all of what ought to be the true standard of the scientific school of medicine. That it requires as extensive preparation in informing, and disciplining the mind, in training the hand and practicing the eyes, that it demands as much purity of character, as high social qualifications, all of that fine, electrical, life-inspiring influence, carrying healing in its mere presence, to adorn and qualify its practitioner, as it does in any other profession, is conceded by all. Hence, the profession of medicine should stand equal to any other calling in civic honor, and public regard. The degree of Doctor of Medicine should be a title of honor, of the highest distinction, all over the land; and every one who dares to take it, should be made to feel that his life was consecrated to the highest uses of society. There should be a divinity to shape the ends of Doctors, rough hew them how they will, and that to the nobler purposes only.

But let me ask, is this the case? Does the title of Doctor of Medicine confer any special regard, giving social position, public estimation above the ordinary level of common life? I am afraid that it does not. Yet many are the individual cases where men have worked out a name and fame that ennoble them, and ought to hallow the calling in the estimation of the world.

Let us examine the status of the medical profession from a few different stand points. and try to calculate what is its true grade of esteem. And, by the "Medical Profession," I most certainly do not intend to include all that rabble of *pathists* denominated Allopathist, Homœopathist, Hydropathist, Eclectic, etc. But that class of practitioners only as represented by our County and State Medical Societies, and the teachers in the leading colleges of medicine.

In this state, under the statute law, they are now on the same level with all quacks, charlatans, mountebanks, empirics, &c. There is no distinction for them. The impostor has the same protection, same privileges, rights and demands. The diploma of our highest schools, in the courts, give no precedence, no higher position to protect the true physician from the false pretender. Now the common law is the outgrowth of the wants and demands of society. It is not the work of a day or of a single mind, but the slow accretion of ages, shaped, altered, and adapted to the desires of the people. Can it be our law-makers finally came to see no difference in those who pracciced the healing art, and thus placed them before the law, on one common level?

Let us take another view of our subject from the standpoint of society at large. Where do the masses of the people go for help in times of sickness? Certainly it is not to those who truly represent the profession. If they did there would not be so many quacks, flaming out with their advertisements, or so many patent medicines sold, warranted to cure anything and everything, but are, in reality, "sovereign cures for empty pockets, and of no peculiar efficacy for any thing else." But let us look closer still. Do we find among the masses any special regard for the regular practitioner, any particular estimation of his worth above the common mountebank? Some may answer, yes. In their extremity, when they become dangerously ill, they go for him then. True. But do they not, in their extremity, when dangerously ill, as often leave him to employ this same quacksalver?

Again the reply may be, this employment of quackery is confined to the lower classes, to the poor and the ignorant. Is it? Do not many of the families of this very city, distinguished for social position, wealth, and even education, employ quackery for their family physician? Is not this the case throughout the land?

Thus we find before the low, among the masses, and among the better classes of society, there is no well established, prevailing, dominant belief in, or recognition of, any superiority of the true medical practitioner over the charlatan and quack. Homœopathy, to-day, has men of the most eminent distinction as its patrons, and yet every truly scientific man, who looks into it, knows it to be a humbug, a false pretense, a mere theory, founded upon one of the hugest absurdities, which its most determined champions do not follow in practice.

That all are interested in this subject of professional standing scarcely needs discussion. Let every individual do what he will, the time will come, in all probability, when he shall need the advice of a physician, and no one can over-estimate how truly his life or happiness may depend upon that advice. How important then, that it should be based upon a true scientific knowledge of his condition. He rightfully demands that there should be no chicanery, no imposture practiced upon him now. Therefore it certainly does follow that no one can have any intelligent desire to encourage, or any true interest in resorting to any false, or doubtful, system of practice. But the larger portion of mankind does, and the law, that leaves them unprotected, is a wrong; and those who induce them to it, should not escape the criminal responsibility of their deeds.

Why is it a profession which, for their important interest, ought to be so sacredly guarded in its practice, so highly reverenced and respected by the people, does, after all, actually possess so little public esteem?

I will endeavor to enumerate a few of what I deem to be prominent causes, which lead to so deplorable a result.

The low standard of qualification required by our schools, which confer the degree of Doctor of Medicine, their total indifference to the moral quality of the character, and the limited general education of their graduates.

The admission of such poorly prepared creatures to the high title and sacred principles of M. D., soon destroys all confidence and prestige in the profession. It lowers its standard at once, and becomes a fruitful source of quackery and imposition, as too many of them strive by such means to make up pecuniarily for what they have not the ability, or time, to honestly and honorably earn. This class of graduates compose far too large a portion of that throng which our medical schools annually turn loose upon the world. They go forth into society, and educate the people in the knowledge of disease and the healing art. Few of them ever study their text books further. The rest of their acquirements are gathered from the world around them, and are taught by sharp experience and surface observation; a school not well adapted to develope a very high standard of scientific knowledge in their profession, but certainly apt to draw them into methods of practice, which lack a high sense of honor, and a fine moral point. They make us think of the poor woman that St. Luke tells of. "A woman having an issue of blood twelve years, which had spent all her living upon physicians, neither could be healed of any."

Are we not sometimes a little undeservedly severe in our comments upon patients of this class, who consult many physicians? Oliver Wendell Holmes quaintly tells of a lady he saw, who, in talking of an illness, told him she had consulted twenty-six different doctors in succession, and was then in search of the twenty-seventh. He says: "I recommended a great master in of the specialties, then residing in the neighborhood, who, I thought would understand her case better than any body else, and she should stick to him and his prescriptions, and give up this butterfly wandering from one chamomile flower of medicine to another."

But why should there be this long search for a physician who can satisfy such cases? Does it not often depend upon the different diagnoses made, the tempting promises held out to effect just such changes? She goes to one of these long-billed physicians, and he tells her "her liver is badly affected." "She is a great sufferer from liver complaint." "That her liver must be attended to at once." "Oh, doctor, can you cure it?" she asks in great trepidatlon over these sad and awful announcements. "Oh, yes, my good woman, most certainly, but it will take a long time, and require a great deal cf medicine." She is now profoundly impressed with her dreadful and precarious condition, but feels a great degree of consolation, and much thankfulness, for finding such a noble, kind hearted and skillful physician, who is so ready to promise her *so* much. How faithfully she swallows the disgusting compounds he gives; and how liberally she pays for them. But this wears out after a while, and being worse rather than better, she seeks No. 2, and learns from him how she has been imposed upon by No. 1. She has no "liver complaint;" it is her heart. "My good woman, your heart is badly affected; you have heart disease, and it should be attended to at once." "Oh, dear, doctor, can you cure it?" "Certainly, but it will take a long time, and require much medicine." The dosing and liberal remuneration are repeated, and she is no better. No. 3 is consulted. "My good woman, your lungs are badly diseased; you have had lung fever, and one lung is nearly gone." "Oh, doctor, is my heart affected too?" "Not at all, madam." "Oh, that scoundrel, how he deceived me; he said I had heart disease." "He was an impostor, madam, and you should not have gone to him." The cure is again promised, and the liberal pay again dealt out. *She still lives*, and is no better, and No. 3 plays out. No. 4 is consulted. Her case, to herself, has now become enveloped in great mystery, and she is pretty thoroughly convinced her disease is "extensive and complicated." No. 4 tells her, after a careful examination of the vital organs, and a history of her case: "Madam, I find your lungs, and heart, and liver are all sound. I do not think there is much the matter with you. If you will lay aside all medicine, be careful in your diet, and take some exercise in the open air daily, you will soon find yourself quite well." This time she is disgusted with the ignorance of doctors. She is offended at being told there is nothing the matter with her. Her feelings too plainly contradict that. No. 4 is a humbug, and she will have nothing more to do with him. But

I forbear. You are too familiar with the history of these cases.

Now, is it difficult to see where the wrong is? That woman believed the story of the first, and of the second, and of the third of these villains, and, becoming bewildered, felt very sure something was seriously the matter with her, and of course she could not believe the truth when it was told to her.

Are the weakness of this class left entirely as game for these leeches? Suppose I should dare to enter the circle of the medical profession, and look a little closely at the practice, which is covered by a medical diploma, might I be a little puzzled to tell where quackery and mountebankery ceased, and the high honor of our noble code of medical ethics began? Would there be any difficulty in discerning whether all that practice, which depends upon boasting one's own cures, and denying the ability of others, whether all the backbiting, slandering, ignoring, insinuating talk lay entirely outside of that circle? In fact where should I be compelled to draw the line which marks true scientific attainment, and high unspotted honor, that now stooped to meanness, from that varying species of quackery known under a hundred kaleidoscopic names?

But it is far easier to criticise than to point out the remedy. "Physician heal thyself!" We all love our noble profession, and would be glad to see it receive its due measure of praise and esteem. We would all rejoice in the purity and honor of its noble brotherhood. Let us then stand united, heart and hand, pledged to a holy support of each other in the cause of justice, and in all works of mercy. The code of medical ethics, that forms our written rule of conduct, is a noble monument both to the head and heart of our profession. Its sentiments should burn in words of living light in the heart of every true member of our band, and shine out in joyous expression in all his conduct.

Suppose there was a community that had a School of Medicine, which was composed only of men who stood foremost in the ranks of true scientific attainment, their minds well stored with the winnowed lore of the past, disciplined to clear and accurate observation, and anxious only for truth, of high moral culture, and a grand, fine sense of honor; determined that their ranks should only be increased by men who had come up to an equal profession of culture, virtue and honor; and equally determined to eliminate any member who might fall from their proud eminence. Suppose they made it part of their life-work to teach the people the pure love of health, and a knowledge of the human system and leading types of disease. Would not such an association rapidly build itself up to the highest attainment in public estimation? Its opinions would carry both weight and conviction with them. Its teaching would soon become the law and gospel of medicine for that community. Quackery, in all its various forms, would die out in that land. The place on the shelf in that community, which has known the patent medicine bottle for so long, would know it no more for ever. True hygienic principles would be disseminated among the masses. Increased happiness would follow. A stronger, better, nobler people would grow up. There would be a gradual development of the race upward toward the source of all Truth; and then would be instituted true scientific progress of the race along the line of the finite towards the Infinite.

"Behold how a little leaven leaveneth the whole lump."

*But where is the leaven?*

